# 

**Published:** 1906-11

**Authors:** 


SUBSCRIPTION $1.00

PUBLISHED MONTHLY.

### ATLANTA JOURNAL-RECORD OF MEDICINE.

NOV. 10 1906

Successor to Atlanta Medical and Surgical Journal, Established 1855, and Southern Medical Record, Established 1870.

VOL. VIII. ATLANTA, GA., NOVEMBER, 1906. No. 8.

